# A Case of the Forgotten Thyroid: The Sequelae of Chronic Untreated Hypothyroidism

**DOI:** 10.7759/cureus.4240

**Published:** 2019-03-12

**Authors:** Melanie Weinstein, Magdalena Pasarica

**Affiliations:** 1 Medical Education and Simulation, University of Central Florida College of Medicine, Orlando, USA; 2 Family Medicine, University of Central Florida College of Medicine, Orlando, USA

**Keywords:** hypothyroidism, radioactive iodine therapy, levothyroxine, thyroid replacement therapy, grave's disease

## Abstract

Hypothyroidism is a common endocrine disorder frequently caused by iodine deficiency, autoimmune disease, or as a result of certain medical treatments such as radioactive iodine. We report a 57-year-old woman 21 years pos-radioiodine ablation therapy for Graves’ disease. She presented to the clinic after more than two decades without medical care with a variety of symptoms, including a left-sided lower facial droop and gait instability, and was found to have bradycardia, hyperlipidemia, and mild depression. After evaluation for vitamin deficiencies, anemia, thyroid dysfunction, and stroke, her symptoms were attributed to iatrogenic hypothyroidism. She was started on appropriate thyroid replacement therapy with subsequent symptom resolution. Patients receiving thyroid destructive therapy require education and close follow-up to prevent the development of severe hypothyroidism and its associated sequelae, which can be easily improved with simple, cost-effective thyroid replacement therapy.

## Introduction

Normal thyroid function is required for the control of metabolism, body temperature, heart rate, menstruation, and other essential body functions. In the United States, up to 13 million people have undiagnosed thyroid failure [1]. Patients who develop hypothyroidism secondary to radioiodine ablation therapy should undergo lifelong serum thyroid stimulating hormone (TSH) screenings every six to 12 months to monitor for the development of hypothyroidism, as recommended by the American Association of Clinical Endocrinologists and the American Thyroid Association [2-3]. Symptoms of thyroid dysfunction often develop insidiously, which can result in delayed presentation, diagnosis, and treatment. Severe symptoms of long-standing thyroid hypofunction include hoarseness, deepening of the voice, weight gain, constipation, xerostomia, cold intolerance, fatigue, puffy eyes, muscle weakness and cramps, menorrhagia, depression, slower cognition, and poor memory. Hoarseness, deep voice, and constipation indicate the highest specificity for hypothyroidism (of 94.5%, 88.5%, and 93.1%, respectively) [1]. Clinical signs and laboratory testing often reveal bradycardia, slowed movement and speech, delayed deep tendon reflex relaxation, edema, hypercholesterolemia, macrocytic anemia, and hyponatremia [4]. However, in current clinical practice, these are rarely seen, as patients are typically diagnosed early and symptoms are few and mild. We present a case of iatrogenic hypothyroidism untreated for over 20 years that offers a window into the chronic sequelae of thyroid failure less commonly encountered in United States clinical practice.

## Case presentation

A 57-year-old Caucasian female, with a past medical history of Graves’ disease of 21 years status post-radioiodine ablation, presented to a free clinic, uninsured, after a 20-year lapse in healthcare. She had increasing anxiety over the past several years regarding her perceived worsening state of health, particularly the recent onset of a left-sided lower facial droop and gait instability. Upon further questioning, the patient also reported palpitations, bilateral arm tingling, scalp tenderness, decreased body hair growth, dry skin, hoarse voice, constipation, difficulty sleeping, fatigue, depression, anxiety, arthralgias, and myalgias. Her past medical history was significant only for Graves’ disease treated with radioiodine ablation. She denied ever taking thyroid replacement medication after her thyroid ablation due to a lack of follow-up from the patient.

**Figure 1:**
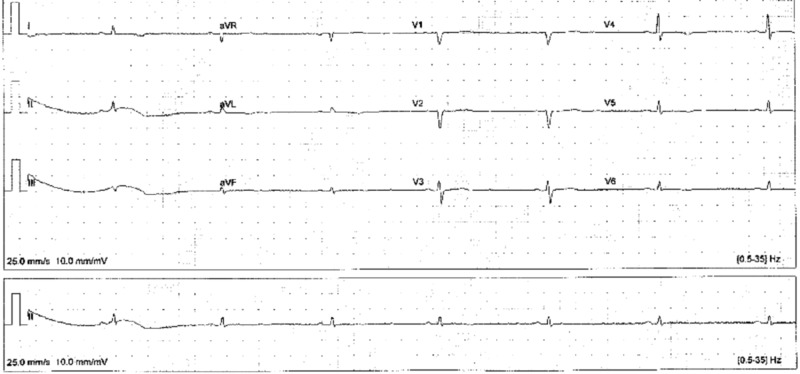
Visit 1 EKG Marked sinus bradycardia (heart rate 44), low voltage, and nonspecific T wave changes EKG: electrocardiogram

On initial presentation, her body mass index (BMI) was 23.3 kg/m^2^, temperature 97.3º F, blood pressure 106/76 mmHg, and pulse 56 beats per minute. Physical exam was remarkable for hoarse voice, bradycardia, dry skin on her hands and legs bilaterally, tender scalp, a left-sided lower facial droop, and anxious affect. Electrocardiogram (ECG) revealed marked sinus bradycardia (heart rate (HR) 44), low voltage, and nonspecific T wave changes (Figure 1).

Patient Health Questionnaire-9 was positive for mild depression. Although the patient’s longstanding untreated hypothyroidism was quickly identified, a comprehensive evaluation was completed to rule out other contributing factors. Serum vitamin B12, folate, and hemoglobin A1C (HbA1c) were within normal limits and a hepatitis panel, rapid plasma reagin (RPR), and stroke work-up were negative. Lipid panel, comprehensive metabolic panel, and complete blood count revealed hypercholesterolemia, elevated liver enzymes, and mild normocytic anemia, respectively (Table 1).

**Table 1:** Blood tests Arrows indicate if serum levels are above or below the normal laboratory range; reference values are listed in parentheses. LDL: low density lipoprotein. WBC: white blood count. Hgb: hemoglobin. MCV: mean corpuscular volume. CR: Creatinine. AST: aspartate aminotransferase.

		Visit 1	Visit 4	Visit 5
Lipid Profile	Cholesterol (100-200 mg/dL)	294 ↑	213 ↑	221 ↑
	LDL (<100 mg/dL)	174 ↑	111 ↑	133 ↑
	Triglycerides (30-150 mg/dL)	132	125	55
CBC	WBC (4.40-10.50 10^3^/uL)	4.4	4.32	
	Hgb (11.4-14.7 g/dL)	11.5 ↓	12.7	
	MCV (80.5-99.8 fL)	99.7	96.6	
CMP	Cr (0.4-1.00 mg/dL)	1.08 ↑	0.65	
	AST (15-41 IU/L)	53 ↑	18	

**Figure 2:**
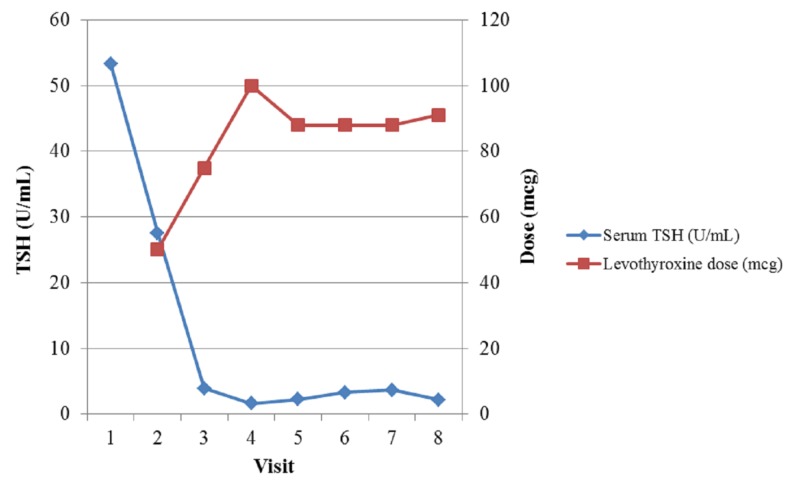
Serum TSH level and levothyroxine dose Clinical evaluation and treatment spanned 20 months Levothyroxine dose for Visit 8 was an average of 91 mcg (88 mcg five days/week, 100 mcg two days/week). TSH: thyroid stimulating hormone

The patient was started on levothyroxine 50 mcg/day and the dose was slowly adjusted according to serial TSH levels (Figure 2).

Although the laboratory TSH reference range was 0.34 U/mL - 5.60 U/mL, the National Academy of Clinical Biochemistry recommended TSH range of 0.4 U/mL - 2.5 U/mL was used due to the patient's age. When the patient’s TSH corrected to within the more stringent target range on levothyroxine 100 mcg/day, she became symptomatic with a tingling sensation in her hands, fatigue, and decreased concentration without any other change in physical examination or medical problems. Her dose was subsequently decreased to 88 mcg/day and her symptoms improved. Furthermore, once she became euthyroid, her cholesterol, low density lipoprotein (LDL), and triglycerides improved while her hemoglobin, creatinine, and aspartate aminotransferase (AST) levels normalized.

**Figure 3:**
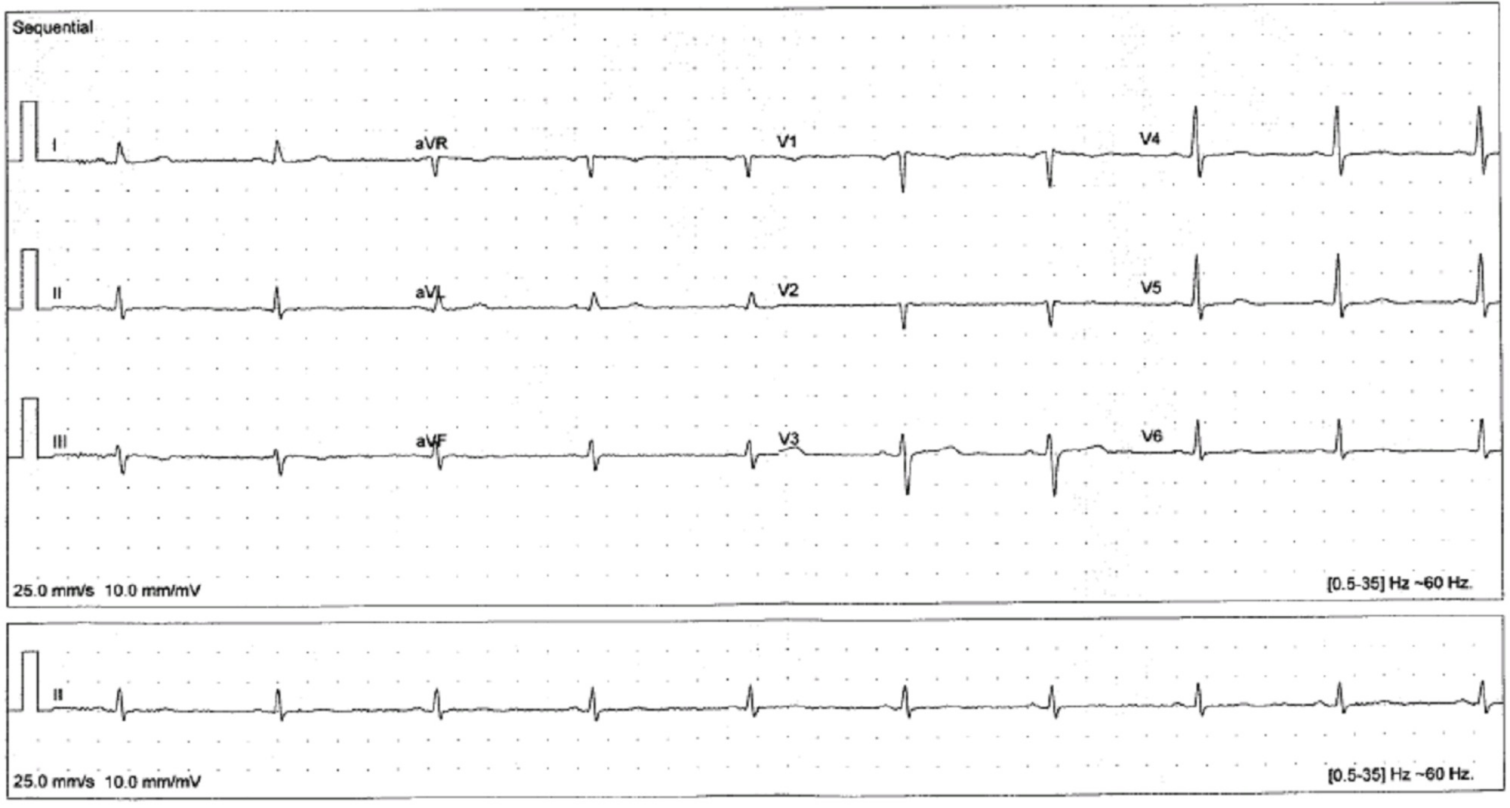
Visit 8 EKG (20 months later) Improved sinus bradycardia (heart rate 58), higher voltage, and nonspecific T wave changes EKG: electrocardiogram

The patient reported improved balance and stability, resolution of her arm tingling and scalp tenderness, increased body hair growth, softer skin, more regular bowel movements, uninterrupted sleep, increased energy, and improved mood. She continued to have myalgias and arthralgias, which were treated conservatively with capsaicin cream and non-steroidal anti-inflammatory medication with clinical improvement. On exam, her voice was less hoarse, heart rate increased, skin appeared normal, facial droop was less prominent, and she was more relaxed. Follow-up ECG showed improved sinus bradycardia and nonspecific T wave changes (Figure 3). After treatment, the patient stated that she felt better than she had in many years. She continues to come to the clinic every six months for TSH monitoring.

## Discussion

The thyroid’s regulatory effect on multiple organ systems leads to a spectrum of signs and symptoms that develop when the gland malfunctions. Through hormonal regulation, the thyroid directs carbohydrate, fat, and protein metabolism as well as cellular oxygen consumption, which, in turn, affects whole body homeostasis [5]. Overall, there is a positive correlation between the degree of TSH deviation and the number of reported symptoms [1].

Autoimmune thyroid disease is the most common cause of thyroid dysfunction in the United States. The incidence of Hashimoto's thyroiditis is approximately 498/100,000 cases of hypothyroidism while Graves’ disease accounts for 99/100,000 of new cases of hyperthyroidism every year [6]. The pathogenesis of both these diseases is T-cell-mediated lymphocytic infiltration of the thyroid tissue, which leads to increased cytokine and autoantibody production that further exacerbates the thyroid dysfunction [7]. Both the American Association of Clinical Endocrinologists and the American Thyroid Association recommend screening serum TSH levels if thyroid dysfunction is suspected. Diagnosis is classified as either overt (elevated TSH and diminished T4) or subclinical (elevated TSH and normal T4) [3]. Overt hypothyroidism affects 1%-2% of the US population while subclinical hypothyroidism affects 4%-10% of the US population, with women affected five to eight times more often than men [1,8]. Spontaneous primary thyroid failure increases in incidence with increasing age for both men and women [9]. Of the 25,000 people screened for a study conducted at the Colorado Health Fair, 9.9% were found to have an untreated TSH abnormality. Many signs and symptoms of hypothyroidism - bowel, skin, and cognitive changes - overlap with those associated with normal aging. The question of who to screen becomes more complicated when approaching the elderly patient [10]. Additionally, screening is recommended for patients with a history of other autoimmune diseases, those who take thyroid-affecting medications, such as lithium or amiodarone, or those with a history of thyroid disease [3].

The treatment of hyperthyroidism includes the use of anti-thyroid medications, thyroidectomy, and radioactive iodine ablation of the thyroid gland. All of these options have the potential to cause hypothyroidism, requiring long-term replacement therapy. In several studies evaluating the efficacy of radioiodine therapy, a minimum of 26% of patients became hypothyroid in the first year, with 2%-3% becoming hypothyroid every year after, up to 87% [2]. The exact percentage of patients who developed hypothyroidism varied based on the dose of radioiodine administered. In line with these, the American Thyroid Association recommends discussing with the patient about the possibility of hypothyroidism after radioactive ablation, the need for thyroid function tests four weeks after the ablation and the need for lifestyle long hormone therapy replacement and/or close follow-up [3].

## Conclusions

This case is significant because it serves as an example of the morbidities that can ensue as a result of a patient being lost to follow-up. It also demonstrates the extent and severity of a patient’s symptomatology given her prolonged hypothyroid state and the importance of treating the underlying cause, not the symptoms. Our patient presented with hypercholesterolemia, bradycardia, facial droop, and mild depression, which could have been due to independent diseases or, as in our case, a direct result of her longstanding untreated hypothyroidism. Additionally, close follow-up and education are essential for patients who receive radioactive thyroid ablation.
